# *Bacillus cereus* Fnr binds a [4Fe-4S] cluster and forms a ternary complex with ResD and PlcR

**DOI:** 10.1186/1471-2180-12-125

**Published:** 2012-06-25

**Authors:** Julia Esbelin, Yves Jouanneau, Catherine Duport

**Affiliations:** 1 Université d’Avignon et des Pays de Vaucluse, UMR408, Sécurité et Qualité des Produits d’Origine Végétale, F-84000, Avignon, France; 2 INRA, UMR408, Sécurité et Qualité des Produits d’Origine Végétale, F-84914, Avignon, France; 3 CEA, DSV, iRTSV, Laboratoire de Chimie et Biologie des Métaux, F-38054, Grenoble, France; 4 UJF-Grenoble 1/CNRS, UMR 5249, F-38041, Grenoble, France

**Keywords:** Fnr, Fe-S cluster, anaerobiosis, *Bacillus cereus*, enterotoxin, DNA binding

## Abstract

**Background:**

Bacillus *cereus* is a facultative anaerobe that causes diarrheal disease in humans. Diarrheal syndrome may result from the secretion of various virulence factors including hemolysin BL and nonhemolytic enterotoxin Nhe. Expression of genes encoding Hbl and Nhe is regulated by the two redox systems, ResDE and Fnr, and the virulence regulator PlcR. *B. cereus* Fnr is a member of the Crp/Fnr family of iron-sulfur (Fe-S) proteins. Only its apo-form has so far been studied. A major goal in deciphering the Fnr-dependent regulation of enterotoxin genes is thus to obtain and characterize holoFnr.

**Results:**

Fnr has been subjected to *in vitro* Fe-S cluster reconstitution under anoxic conditions. UV-visible and EPR spectroscopic analyses together with the chemical estimation of the iron content indicated that Fnr binds one [4Fe-4S]^2+^ cluster per monomer. Atmospheric O_2_ causes disassembly of the Fe-S cluster, which exhibited a half-life of 15 min in air. Holo- and apoFnr have similar affinities for the *nhe* and *hbl* promoter regions, while holoFnr has a higher affinity for *fnr* promoter region than apoFnr. Both the apo- and holo-form of Fnr interact with ResD and PlcR to form a ternary complex.

**Conclusions:**

Overall, this work shows that incorporation of the [4Fe-4S]^2+^ cluster is not required for DNA binding of Fnr to promoter regions of *hbl* and *nhe* enterotoxin genes or for the formation of a ternary complex with ResD and PlcR. This points to some new unusual properties of Fnr that may have physiological relevance in the redox regulation of enterotoxin gene regulation.

## Background

*Bacillus cereus* is a facultative anaerobic bacterium that can cause two types of food-borne illness in humans. Among these, the diarrheal syndrome may result from the production in the human host’s small intestine of various extracellular factors including hemolysin BL (Hbl) and nonhemolytic enterotoxin Nhe [1,2]. The genes encoding Hbl and Nhe belong to the PlcR regulon [3]. The ability of *B. cereus* to produce enterotoxins and grow well in an O_2_-limited environment such as that prevailing in the human small intestine is controlled by both the two-component system ResDE and the redox regulator Fnr. Unlike ResDE, Fnr is essential for *B. cereus* growth under anaerobic fermentative conditions and for *hbl* and *nhe* expression, irrespective of the oxygenation conditions [4,5]. *B. cereus* Fnr is a member of the large Fnr/Crp superfamily of transcription factors that bind as homodimers to palindromic sequences of DNA, each subunit binding to one half-site [6]. Like its homologue from *Bacillus subtilisB. cereus* Fnr contains a C-terminal extension with four cysteine residues, C(x_4_)C(*x*_2_)C(x_3_)C. The last three cysteine residues were identified as [4Fe-4S]^2+^ cluster ligands in *B. subtilis* Fnr, the fourth ligand being an aspartate residue [7]. The integrity of this oxygen-labile Fe-S cluster was found to be essential for the DNA binding activity of *B. subtilis* Fnr [7,8]. By contrast, *B. cereus* Fnr appeared active in DNA-binding protein in its apo-form (cluster-free form). This has led to the conclusion that unlike its *B. subtilis* homologue, *B. cereus* Fnr is active in both its apo-form and its holo-form (bearing a Fe-S cluster) [9]. However, data evidencing that *B. cereus* Fnr could coordinate a Fe-S cluster under anaerobiosis were lacking.

Here, we show that purified *B. cereus* apoFnr can bind one [4Fe-4S]^2+^ cluster per monomer upon incubation with iron, cysteine and cysteine desulfurase. Reconstituted Fnr (also referred to as holoFnr) showed enhanced DNA binding activity within the *fnr* promoter, but no activity difference with regard to the *hbl* and *nhe* promoters. Both the apo- and holo-form of Fnr interact with ResD and PlcR to form a ternary complex. Our results lend novel insight into the molecular control of enterotoxin gene expression in anaerobically-grown *B. cereus*.

## Results

### *B. cereus* apoFnr binds a labile [4Fe-4S]^2+^ cluster

**Figure 1:**
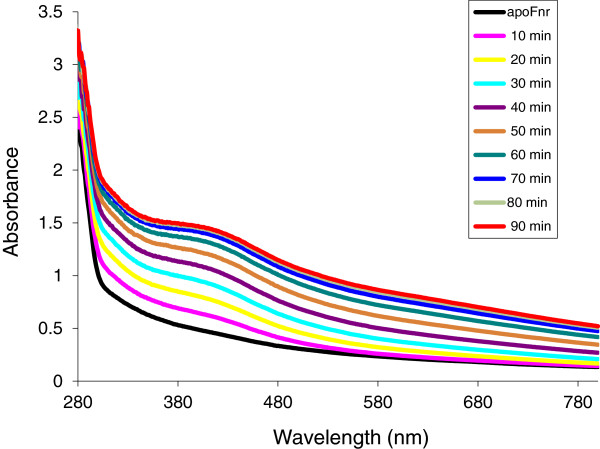
**UV-visible spectroscopy of *B. cereus* Fnr Fe-S cluster reconstitution.** Reconstitution was carried out inside an anaerobic glove box as described in Methods. Time points at which samples were scanned by a UV-visible spectrophotometer are indicated.

*B. cereus* Fnr was expressed as a tag-less polypeptide in aerobically-grown *E. coli* and purified in three steps as described in Methods. The *M*_r_ of the purified polypeptide, as estimated by SDS-PAGE under reducing conditions (with DTT) was 25,000, consistent with the theoretical value of 25,640 deduced from the DNA sequence (Additional file 1). The apparent molecular mass of recombinant Fnr, as determined by analytical gel filtration chromatography and by SDS-PAGE under non-reducing conditions (no DTT or β-mercaptoethanol), was *ca.* 60,000, indicating that tag less Fnr occurs mainly as a dimer in solution. As isolated, the Fnr protein was colorless, contains no detectable iron and its UV-visible spectrum did not feature any absorption band other than that at 280 nm (Figure 1). Thus, we have successfully purified a recombinant tag-less dimeric apo-form of Fnr that is amenable to further investigation *in vitro*.

**Figure 2:**
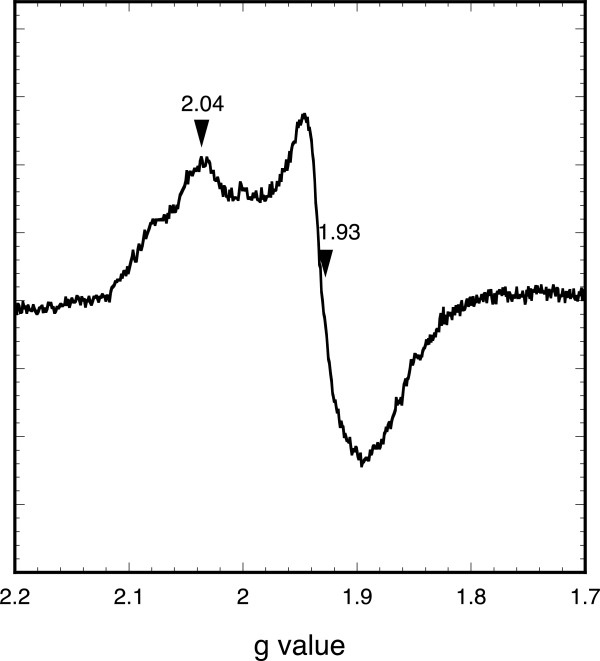
**EPR spectrum of *B. cereus* holoFnr after reduction with dithionite.** The spectrum was acquired under the following conditions: microwave power 0.1 mW, modulation amplitude 1 mT, receiver gain 2.10, temperature 10 K. Relevant *g* values are indicated.

The ability of apoFnr to bind an iron-sulfur cluster under anaerobiosis was tested in an enzyme-driven reconstitution system involving the cysteine desulfurase (CsdA) from *E. coli* (see details in *Methods*). During anaerobic reconstitution, a brown colour developed resulting from a time-dependent increase of a broad absorbance band around 416 nm, typical for [4Fe-4S] containing proteins (Figure 1). After 90-min reconstitution and subsequent gel filtration, the purified brown-colored protein displayed an *A*_416_/*A*_280_ ratio of 0.33 and was found to contain 3.6 ± 0.1 moles of iron atoms per mole of monomer. These data are consistent with the reconstitution of one [4Fe-4S] cluster per Fnr monomer [8,10]. The reconstituted holoFnr was analysed by EPR spectroscopy, both as isolated and after reduction with sodium dithionite. No signal was detected for the Fnr sample as isolated, but a broad signal with main *g* values at 2.04, 1.93 was observed upon reduction (Figure 2). These data indicate the presence of a [4Fe-4S]^2+^cluster, which upon one-electron reduction, converted to a paramagnetic [4Fe-4S]^1+^ cluster with an electronic spin S = 1/2. However, the EPR signal differed from that of typical [4Fe-4S] proteins in that the resonance lines were relatively broad and showed additional features, especially at high field. As a consequence of this broadening, the *g*_x_ component of the tensor was not well resolved. This might reflect some heterogeneity in the vicinity of the cluster, and could be related to the instability of holoFnr upon reduction (see below). In addition, the intensity of the EPR signal was low compared to the protein concentration, although we could not give an accurate estimation of the electronic spin due to the broadening and weakness of the signal. This suggested that the protein was partially reduced, consistent with the observation that dithionite reduction caused a relatively small decrease of the chromophore absorption (data not shown). Attempts to further reduce the protein by using photoreduced 5-deazaflavin were unsuccessful, likely because of the instability of the cluster in the reduced state (data not shown). Taken together, these results suggest that holoFnr contains a redox-responsive [4Fe-4 S] cluster, which is unstable upon reduction.

**Figure 3:**
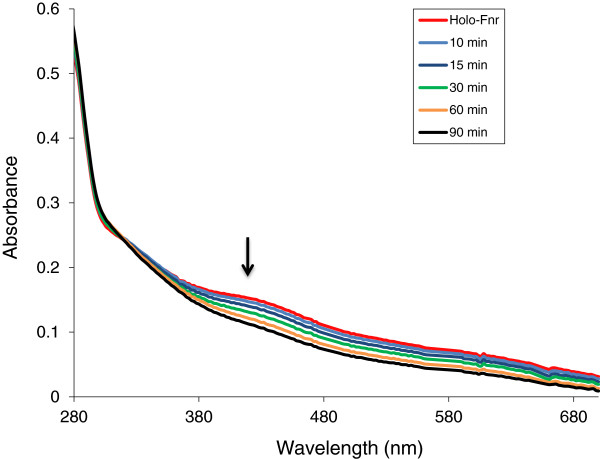
**Changes in the ultraviolet/visible spectrum of reconstituted *B. cereus* Fnr in response to O**_**2**_**.** Spectra of *B. cereus* holoFnr [0.56 g/L] were recorded before and 10 min, 15 min, 30 min, 60 min after exposure to oxygen. Arrow indicates the trend of the spectral changes.

Exposure of reconstituted holoFnr to air resulted in decreased intensity of the 416 nm absorption band associated with the [4Fe-4 S] cluster over 60 min (Figure 3). Based on the absorbance decay at 416 nm, which followed first-order kinetics, the half-life of holoFnr in air was estimated to be 15 min. We conclude that the [4Fe-4S]^2+^ cluster of holoFnr was extremely oxygen-labile.

### DNA-binding properties of *B. cereus* holoFnr

**Figure 4:**
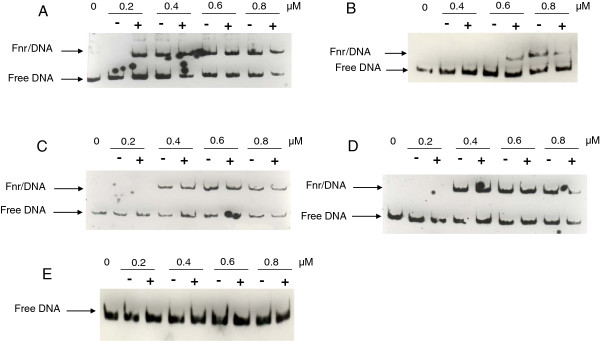
**Binding of apo- and holoFnr to promoter regions of *fnr*, *hbl* and *nhe* genes determined by EMSA.** DNA probes corresponding to *fnr* (**A**), *nhe* (**B**), *hbl1* (**C**), *hbl2* (**D**) and a negative control (**E**) were bound with increasing concentrations of apoFnr (−) and holoFnr (+) as indicated. The results are representative of triplicate experiments.

The DNA-binding properties of holoFnr were investigated with electrophoretic mobility shift assays (EMSA) under strict anoxic conditions. Figure 4 shows the EMSA results obtained using holo- and apoFnr and the promoter regions of *fnr* (Figure 4A), *nhe* (Figure 4B) and *hbl*. Because of its large size (1,157 bp), the promoter region of *hbl* was divided into two overlapping fragments of 636 bp (*hbl1*, Figure 4C) and 610 bp (*hbl2*, Figure 4D). The binding specificity was evidenced from the disappearance of the complexes in competition assays using a 50-fold excess of homologous unlabelled promoter regions (data not shown) and by the absence of binding with the negative control (Figure 4E). The results showed that (i) all the complexes formed were stable and did not dissociate during electrophoresis, (ii) the presence of the [4Fe-4S]^2+^ cluster increased Fnr-binding affinity to *fnr* and *nhe* promoter regions and did not affect Fnr-binding to *hbl* promoter regions. Regarding the *nhe* promoter, the observed difference in apparent binding affinity between the apo- and holo- forms was narrow (≤ 1.3). Also, a fairly high level of Fnr (more than 0.6 μM) was needed to form the DNA-Fnr complex. These data suggest that holo- and apoFnr have similar affinities for the *nhe* promoter.

### Fnr forms a ternary complex with ResD and PlcR

**Figure 5:**
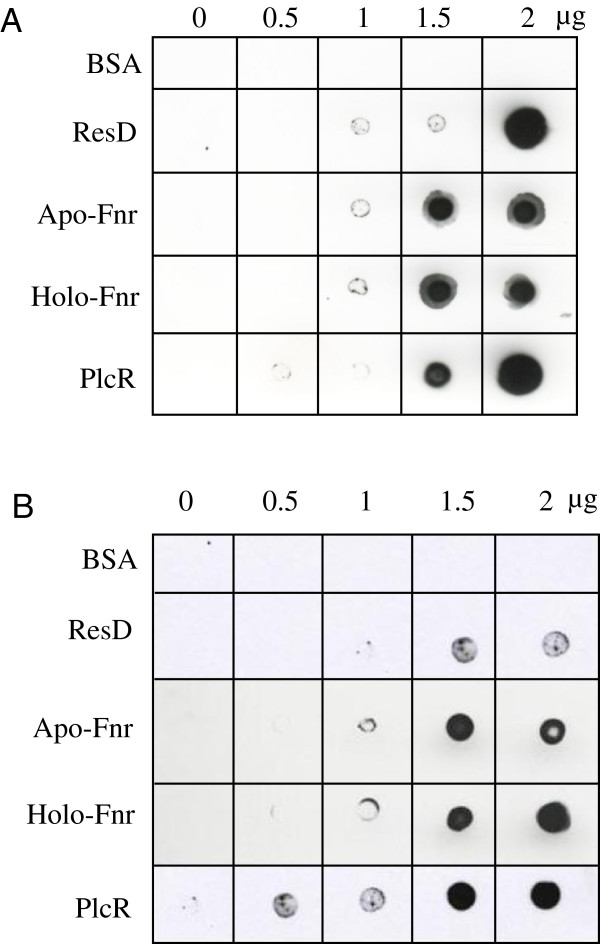
**Far-Western analysis of PlcR-Fnr, PlcR-ResD and ResD-Fnr interactions.** Increased amounts of purified Fnr, ResD and PlcR were spotted onto nitrocellulose membranes and incubated with biotinylated-PlcR (**A**) or biotinylated-ResD (**B**), under anoxic conditions. PlcR and ResD binding was detected using streptavidin-HRP complex and visualized by chemiluminescence. BSA was used as negative control.

To determine whether Fnr could interact *in vitro* with PlcR and ResD, two other regulators of *nhe* and *hbl*, Far-Western analyses were conducted under anoxic conditions using the apo- and holo- forms of Fnr. Figure 5 shows that (i) BSA (negative control) did not bind to PlcR or ResD, while PlcR and ResD showed self-binding consistent with their capacity to oligomerize [11,12], (ii) both apo- and holoFnr interact with PlcR and ResD and (iii) PlcR interacts with ResD. These pairwise interactions were confirmed by cross-linking experiments using dimethyl suberimidate (Additional file 2).

**Figure 6:**
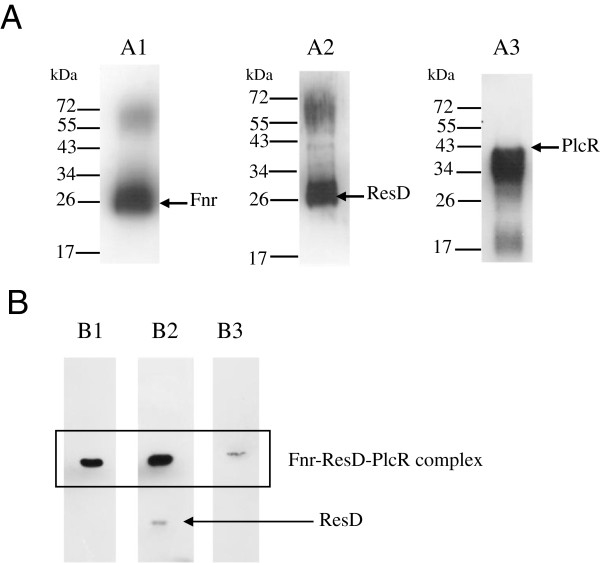
**Western blot analysis of proteins from *B. cereus* crude extract immunoprecipitated with immobilized Fnr-specific antibodies.** (**A**) Proteins resulting from an anti-Fnr pull-down were analyzed by Western blotting with anti-Fnr (**A1**), anti-ResD (**A2**) or anti- PlcR (**A3**) antibodies. (**B**) Immunoblot analysis of the same protein samples after non-denaturing electrophoresis (10% native gel) using anti-Fnr (**B1**), anti-ResD (**B2**) or anti-PlcR (**B3**) antibodies.

To determine whether Fnr could interact *in vivo* with PlcR and ResD, soluble protein extracts were prepared from anaerobically-grown *B. cereus* cells and incubated with anti-Fnr antibodies. Figure 6A shows that anti-Fnr antibodies could co-precipitate ResD and PlcR independently. Interestingly, Figure 6B shows that anti-Fnr antibodies co-immunoprecipitated ResD, PlcR and Fnr. These results strongly suggest that Fnr, ResD and PlcR form a ternary complex *in vivo*.

## Discussion

This work has shown that the Fnr protein of *B. cereus* is homodimeric and can bind one [4Fe-4 S] iron-sulfur cluster per monomer. Our first challenge was to accurately assemble the Fe-S cluster via an enzymatic system since all our attempts to purify holoFnr under anaerobiosis failed. We demonstrated that CsdA from *E. coli* was capable of assembling the *B. cereus* Fnr Fe-S cluster. Interestingly, *B. cereus* synthesizes [13]one pyridoxal 5-phosphate-containing enzyme (NP_834652) [13] that might be involved in Fe-S cluster biogenesis. When anaerobically reconstituted *B. cereus* Fnr was exposed to O_2_, we observed a rapid loss of the Fe-S cluster, demonstrating that Fnr functions as an oxygen sensor via its Fe-S cluster. Importantly, the cluster of the reconstituted *B. cereus* Fnr appeared extremely unstable, judging from its fast destruction on exposure to air. In this respect, the *B. subtilis* holoFnr, which is the closest homolog of *B. cereus* Fnr [14] displayed greater stability [8]. Sequence comparison of the *B. cereus* and *B. subtilis* Fnr revealed a significant variation in the amino acid residues around the three C-terminal cysteine residues (C219-*X*_2_-C222-X_4_-C227) that serve as ligands for the cluster (Additional file 3) [7]. These observations imply that the occurrence of certain amino acid residues close to the cluster ligands may affect the stability of the *B. cereus* holoFnr, thus providing a possible explanation for its high susceptibility to oxygen damage [15]. As a result, *B. cereus* Fnr might sense subtle changes in the redox status of the cells, a property that would reflect an adaptation of the pathogenic strain to the environment of its human host.

We proposed previously that *B. cereus* apoFnr binds promoter regions of enterotoxins only through the monomer pathway. In other words, we proposed that apoFnr was active as a DNA-binding protein only under its monomeric form [9]. Here we showed that, when produced in a tag-less form, apoFnr is active as a DNA binding protein under its dimeric form. In addition, we showed that dimeric apoFnr-DNA complexes were stable in contrast to what we observed previously [9]. We conclude that (i) in our previous studies, tags fused at the N-terminus and C-terminus of Fnr introduced steric hindrance that affected its oligomeric structure and/or DNA binding activity and (ii) *B. cereus* apoFnr may bind DNA both through the dimer and the monomer pathway under aerobiosis unlike its homologues of *B. subtilis* and *E. coli*[8]. There are probably many variables affecting the choice for a monomer or dimer recognition pathway *in vivo*. Among them, there is the redox state of the cell that may impact directly the ratio of monomeric to dimeric apoFnr since we observed that the addition of reductant (DTT) affected the dimerization state of apoFnr in solution. Finally, the mechanism of apoFnr dependent regulation of enterotoxin is undoubtedly complex, and further extensive experiments are required to examine the role of the monomer and dimer pathways.

**Figure 7:**
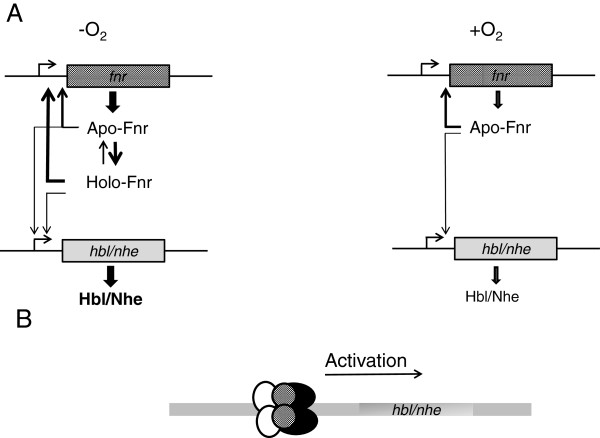
**Proposal for the Fnr-dependent regulation of the *hbl* and *nhe* enterotoxin genes in *B. cereus.*** (**A**) apo- and holoFnr-dependent regulation in either the absence or presence of oxygen. (**B**), Fnr is thought to be part of a ternary complex involving ResD (black), PlcR (white), Fnr (gray), acting as positive regulator.

The properties of the Fe-S cluster indicate that Fnr is essentially present in the apo- form in aerobically grown *B. cereus,* and may occur in both apo- and holo- forms in anaerobically-grown bacteria, the ratio between the two forms depending on the redox status of the cells, as detected by the Fnr cluster (Figure 7). The stability of the holo form might also be modulated through interactions with DNA, protein partners and (or) low-molecular weight thiols [16-18]. Given the higher DNA binding affinity of the holo form compared with the apo form to its own promoter, we assume that higher levels of Fnr (apo + holo) are produced under anaerobiosis than under aerobiosis (Figure 7). In addition, on the basis of these and earlier results, we offer evidence that Fnr can (i) activate the expression of genes encoding the enterotoxin-activators *resD* and *plcR* and (ii) associate with PlcR and ResD to form a ternary complex under both anaerobiosis and aerobiosis [4,5,9,11]. By producing higher levels of Fnr [5], anaerobically-grown *B. cereus* cells might produce higher levels of the tripartite Fnr-ResD-PlcR complex and, as a result, higher levels of Hbl and Nhe. Hence, the interconversion between apo- and holoFnr may well be a key factor in controlling the regulation of enterotoxin gene expression through the Fnr/PlcR/ResD complex.

## Conclusions

In conclusion, this work brings further evidence that *B. cereus* Fnr, unlike its counterpart from *B. subtilis*, is an active transcriptional regulator in both its apo- and holo- forms. This property may enable *B. cereus* to ensure optimal enterotoxin gene expression in response to changes in oxygen tension such as those encountered during infection of the human host.

## Methods

### Bacterial strains and growth conditions

*Escherichia coli* strain TOP10 (Invitrogen) was used as the general cloning host, and strain BL21 CodonPlus(DE3)-RIL (Stratagene) was used to overexpress *fnr* and *resD*. *E. coli* strain BL21λDE3, containing the pRep4 plasmid [19] was used to overexpress *plcR*[12]. *E. coli* strains were routinely grown in Luria broth at 37°C.

### Recombinant expression of *fnr*, *resD* and *plcR* and protein purifications

The coding sequence for *B. cereus fnr* was PCR amplified from F4430/73 genomic DNA using primers PET101F (5'-CACCATGACATTATCTCAAG-3') and PET101R (5'-CTAATCAATGCTACAAACAGAAGC-3'). The amplicon was cloned as a blunt-end PCR product into pET101/D-TOPO (Invitrogen), yielding pET101*fnr*. *B. cereus* Fnr was produced as a recombinant protein in aerobically grown *E. coli* BL21(pET101*fnr*). The culture was grown in a 6-litre fermentor until OD_600_ reached ~1.0, and protein overproduction was then triggered by 0.2 mM isopropyl-ß-d-thiogalactopyranoside (IPTG). After incubation for 16 h at 20°C, cells were harvested by centrifuging at 10,000 × *g* for 15 min. Fnr was then purified as follows: the bacterial pellet was resuspended in 120 ml of buffer C (25 mM Tris–HCl [pH 8], 1 mM dithiothreitol (DTT)) and incubated with 0.2 mg.ml^-1^ of lysozyme and 5 mM EDTA for 10 min at 30°C. Cells were lysed by ultrasonication for 3 min using a Vibra cell ultrasonifier (Fisher Bioblock Scientific). Cell debris and membrane particles were removed by centrifuging at 43,000 × *g* for 1 h, and the resulting supernatant was loaded on a 30 ml DEAE-cellulose column (DE52; Whatman) equilibrated with buffer C. The non-retained fraction was adjusted to pH 7 with 1 M KH_2_PO_4_ and then loaded onto a 20 ml hydroxyapatite column (HA Ultrogel; Pall Corporation) equilibrated with buffer D (50 mM KH_2_PO_4_ [pH 7], 1 mM DTT). The column was developed with a linear gradient from 50 to 200 mM KH_2_PO_4_ at a flow rate of 2 ml/min. Fractions containing recombinant Fnr were pooled and concentrated by ultrafiltration through an Omega disc membrane (30 kDa cutoff, Ø 43 mm, Pall Corporation). A polishing step was then carried out by gel filtration on a column of Superdex SD200 (1.5 × 60 cm, Amersham Biosciences) equilibrated with buffer F (25 mM Tris–HCl [pH 7.5]1, 50 mM NaCl, 1 mM DTT). The purified protein, >90% pure by sodium dodecyl sulfate-polyacrylamide gel electrophoresis (SDS-PAGE; Additional file 1), was stored as pellets in liquid nitrogen.

Recombinant expression and purification of *resD* and *plcR* were performed using previously described methods [11,12].

### Reconstitution of Fnr holoprotein

The following procedure was carried out under anoxic conditions (O_2_ < 1 ppm) in a glove box maintained at 18°C (Jacomex, France). All buffers were degassed under argon and equilibrated for at least 16 h in the glove box before use. ApoFnr (2 g/L) was incubated with 1 μM cysteine desulfurase CsdA from *E. coli*[20], 1 mM l-cysteine, and 1 mM Fe(NH_4_)_2_(SO_4_)_2_ (Sigma-Aldrich) in the presence of 4 mM DTT in buffer F. Formation of the cluster was monitored by UV-visible spectroscopy using a Uvikon spectrophometer (Kontron) connected through optic fibers to the cuvette holder inside the glove box. The reaction was initiated by adding CsdA, and reached completion after 2 h (no further increase in the absorption at 416 nm). The protein was run through a 10 ml Sephadex G25 column (Amersham Biosciences) equilibrated in buffer F to remove excess reagents, and then concentrated by ultrafiltration using a Nanosep device with a molecular cutoff of 30 kDa (Pall Corporation).

### Protein biochemical analyses

Protein concentrations were determined by either a bicinchoninic acid (BCA) assay (Pierce) or a Biuret method insensitive to thiols [21]. Bovine serum albumin (BSA) was used as a standard. The presence of the Fnr protein in purification fractions was monitored by SDS-PAGE [22], followed by Coomassie blue staining. The iron content of holoFnr was determined spectrophotometrically using a method adapted from Blair and Diehl [23]. Briefly, 50 μl samples of holoFnr (2.8 g/L) were incubated at 100°C for 15 min with 30 μL of 6 N HCl. After dilution to 0.5 ml with H_2_O, samples were centrifuged at 12,000 × *g* for 5 min, and 100 μl aliquots of the supernatant fractions were mixed with 0.65 ml of 0.5 M Tris–HCl pH 8.5, 50 μl of 5% ascorbate and 0.2 ml of 0.1% bathophenanthroline (Sigma-Aldrich). Mixtures were incubated at room temperature for 1 h, and the absorbance was measured at 536 nm (*ϵ*_536_ = 22.14 mM^-1^ cm^-1^) and compared with a blank lacking holoFnr.

### Spectroscopic characterization of holoFnr

Samples were prepared in an anaerobic glove box at 18°C. HoloFnr (0.1 mM) was tentatively reduced with 10 μM 5-deazaflavin (a gift from Prof J. Knappe, Heidelberg University, Germany) in the presence of 2.5 mM glycine as electron donor. Photoreduction was carried out in a 0.2 cm light path cuvette by exposing the protein sample to the light of a slide projector for 1 min time periods. Chemical reduction was also applied with an excess of sodium dithionite (2 mM) at pH 8.5. Progression of the reaction was monitored by recording UV-visible absorption spectra in the 300–700 nm range.

Samples were transferred into EPR tubes and immediately frozen in liquid nitrogen. EPR spectra were recorded at 10 K using a Bruker EMX spectrometer equipped with an Oxford Instruments ESR900 liquid helium cryostat.

To assess the sensitivity of holoFnr to oxygen, a fraction of the reconstituted protein was removed from the glove box and exposed to air. Absorbance spectra were recorded at time intervals with an HP8452 diode-array spectrophotometer (Agilent).

### *Protein-protein interactions*

*Far-Western assays and cross-linking reactions were carried out in an anaerobic glove box as described previously [*[9]*]. Revelation in Far-Western assays used biotinylated PlcR or biotinylated ResD. The cross-linked products were analyzed by 12% SDS-PAGE and detected by Western blotting using anti-Fnr and anti-ResD antibodies.*

### Anaerobic electrophoretic mobility gel shift assay (EMSA)

EMSAs were performed in an anaerobic glove box. Fragments containing the promoter regions of *fnrhbl*, and *nhe* were PCR-amplified and end-labeled with the following biotinylated primer pairs: FnrFbiot (5'-CGAACACTTCAGCAGGCATA-3') and FnrR (5'-AATGTCATACTGTTTGCCAC-3'), Hbl1Fbiot (5'-GGTAAGCAAGTGGGTGAAGC-3') and Hbl1R (5'-AATCGCAAATGCAGAGCACAA-3'), Hbl2Fbiot (5'-TTAACTTAATTCATATAACTT-3') and Hbl2R (5'-TACGCATTAAAAATTTAAT-3'), NheFbiot (5'-TGTTATTACGACAGTTCCAT-3') and NheR (5'-CTGTAACCAATAACCCTGTG-3'), respectively. DNA fragment used as negative control was part of sequence BC0007 (NC_004722) and was amplified with the biotinylated primer pairs: F16biot (5’-GGTAGTCCACGCCGTAAACG-3’) and R16 (5’-GAAAACCATGCACCACCTG-3’). The 5’-labeled amplicons were purified using the High Pure PCR Product Purification Kit (Roche). Binding reactions were performed for 30 min at 37°C by incubating biotin-labeled DNA fragments (2 nM per reaction) with the indicated amount of purified apo- or holoFnr (0.2, 0.4, 0.6 and 0.8 μM) in 10 mM Tris–HCl [pH 7.5] buffer containing 50 mM KCl, 1 mM DTT, 2.5% glycerol, 5 mM MgCl_2_ and 5 mg/L of poly(dI-dC). The samples were resolved by electrophoresis on a 6% non-denaturing polyacrylamide gel [9] and electrotransferred onto Nylon membranes (Amersham Hybond N+). Biotin-labeled DNAs were detected using the LightShift Chemiluminescent EMSA Kit (Pierce).

### Co-immunoprecipitation

*B. cereus* F4430/73 protein lysates were prepared as follows: anaerobically-grown cells were harvested by centrifuging, washed twice with phosphate-buffered saline (PBS; 0.14 M NaCl, 2.68 mM KCl, 10.14 mM Na_2_HPO_4_, 1.76 mM KH_2_PO_4_ [pH 7.4]), resuspended in lysis buffer (10 mM Tris, 1 mM EDTA, [pH 8]), and mechanically disrupted using a FastPrep instrument (FP120; Bio101, Thermo Electron Corporation). Cell debris were removed by centrifuging (3500 × *g*, 10 min, 4°C). The protein lysate was then filtered through a 0.22 μm membrane; 100 μl of cleared lysate was incubated with 50 μl of anti-Fnr protein A-coated Dynabeads prepared by mixing 50 μl of polyclonal anti-Fnr [11] with 50 μl of protein A Dynabeads (Dynal). The beads were pelleted by centrifuging, washed three times with PBS buffer, and suspended in 20 μl of loading buffer. Samples were either directly analyzed by non-denaturing PAGE, or boiled and subjected to 12% SDS-PAGE. Resolved proteins were transferred to a nitrocellulose membrane (Amersham Bioscience) according to standard procedures (Bio-Rad). Membranes were probed with 1:2,000, 1:1,000 and 1:2,000 dilution of polyclonal rabbit sera raised against Fnr, ResD and PlcR, respectively [9,11,24]. The blotted membranes were developed with 1:2,000 dilution of goat anti-rabbit IgG peroxidase-conjugate (Sigma-Aldrich) and an enhanced chemiluminescence substrate (Immobilon Western, Millipore).

## Abbreviations

Hbl: hemolysin BL; Nhe: non-hemolytic enterotoxin; EMSA: Electrophoretic Mobility Gel Shift Assay.

## Competing interests

The authors declare that they have no competing interests.

##  Authors’ contributions

JE performed experiments, developed and performed analyses and assays, analyzed the data and contributed to the writing. YJ and CD designed the research, discussed the results and wrote the paper. All authors read and approved the final manuscript.

## Supplementary Material

Additional file 1 **Figure S1.** SDS-PAGE analysis of overproduced and purified *B. cereus *Fnr. Samples of the purification fractions were analyzed by electrophoresis on an reducing SDS-12% polyacrylamide gel followed by Coomassie Brillant Blue staining. The position and mass (kDa) of molecular weight markers (lanes 1) are given on the left. Lane 1, standard proteins. Lane 2, soluble whole cell extract from *E. coli*. Lane 3, DE52 flow-through. Lane 4, hydroxyapatite pool. Lane 5, purified protein after Superdex 200 size exclusion chromatography. Click here for file

Additional file 2 **Figure S2.** Western Blot analysis of the cross-linked products between Fnr, ResD and PlcR. Proteins were visualized by immunoblotting with anti-Fnr (A) or anti-ResD antibodies (B). (A) Lane 1: untreated Fnr; Lane 2: Fnr preincubated with DMS, Lane 3: Fnr and ResD preincubated with DMS; Lane 4: Fnr and PlcR preincubated with DMS.(B) Lane 1: untreated ResD; Lane 2: ResD preincubated with DMS, Lane 3: ResD and PlcR preincubated with DMS. Click here for file

Additional file 3 **Figure S3.** Sequence analysis of *B. cereus *Fnr. Sequence alignment was performed using ClustalW software. Conserved residues are indicated by a star; conservatively substituted residues are indicated by a colon and semi-conservatively substituted residues are indicated by a point. The cysteine residues are indicated in bold. The cysteine residues 227, 230 and 235 that coordinate the [4Fe-4S]^2+^ cluster with aspartate residue 141 in *B. subtilis* are indicated in gray. Click here for file
