# Supplementary material for: Bacillus cereus Fnr binds a [4Fe-4S] cluster and forms a ternary complex with ResD and PlcR
Source: BMC Microbiol. 2012 Jun 25;12:125. doi: 10.1186/1471-2180-12-125 (PMC3520743; doi:10.1186/1471-2180-12-125)
Supplement: Additional file 1 — Figure S1. SDS-PAGE analysis of overproduced and purified B. cereus Fnr. Samples of the purification fractions were analyzed by electrophoresis on an reducing SDS-12% polyacrylamide gel followed by Coomassie Brillant Blue staining. The position and mass (kDa) of molecular weight markers (lanes 1) are given on the left. Lane 1, standard proteins. Lane 2, soluble whole cell extract from E. coli. Lane 3, DE52 flow-through. Lane 4, hydroxyapatite pool. Lane 5, purified protein after Superdex 200 size exclusion chromatography. [file 1471-2180-12-125-S1.pptx]

## Slide 1
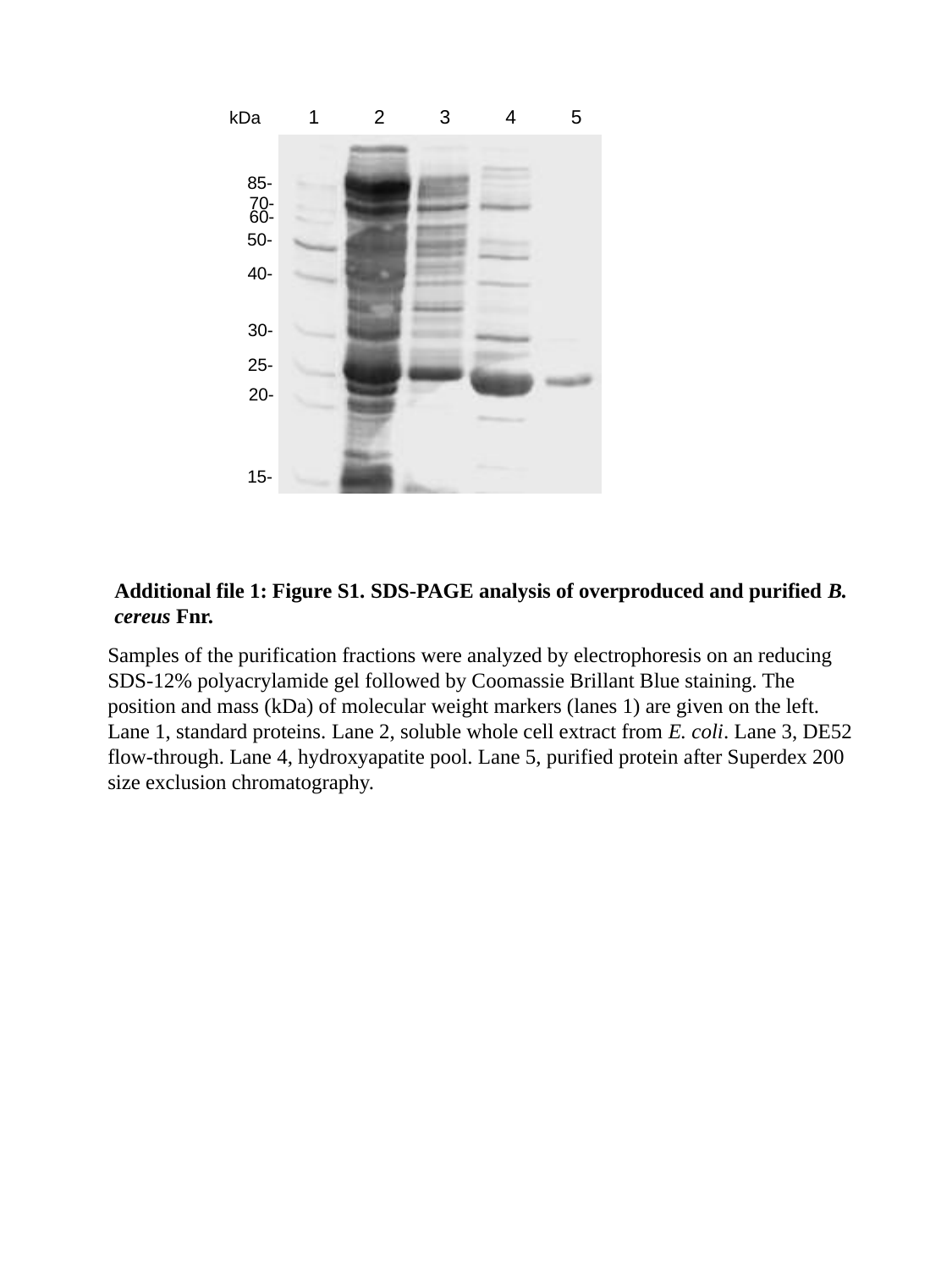

1 2 3 4 5
kDa
85-
70-
60-
50-
40-
30-
25-
20-
15-
Additional file 1: Figure S1. SDS-PAGE analysis of overproduced and purified B. cereus Fnr.
Samples of the purification fractions were analyzed by electrophoresis on an reducing SDS-12% polyacrylamide gel followed by Coomassie Brillant Blue staining. The position and mass (kDa) of molecular weight markers (lanes 1) are given on the left. Lane 1, standard proteins. Lane 2, soluble whole cell extract from E. coli. Lane 3, DE52 flow-through. Lane 4, hydroxyapatite pool. Lane 5, purified protein after Superdex 200 size exclusion chromatography.
