# Supplementary material for: Bacillus cereus Fnr binds a [4Fe-4S] cluster and forms a ternary complex with ResD and PlcR
Source: BMC Microbiol. 2012 Jun 25;12:125. doi: 10.1186/1471-2180-12-125 (PMC3520743; doi:10.1186/1471-2180-12-125)
Supplement: Additional file 2 — Figure S2. Western Blot analysis of the cross-linked products between Fnr, ResD and PlcR. Proteins were visualized by immunoblotting with anti-Fnr (A) or anti-ResD antibodies (B). (A) Lane 1: untreated Fnr; Lane 2: Fnr preincubated with DMS, Lane 3: Fnr and ResD preincubated with DMS; Lane 4: Fnr and PlcR preincubated with DMS.(B) Lane 1: untreated ResD; Lane 2: ResD preincubated with DMS, Lane 3: ResD and PlcR preincubated with DMS. [file 1471-2180-12-125-S2.pptx]

## Slide 1
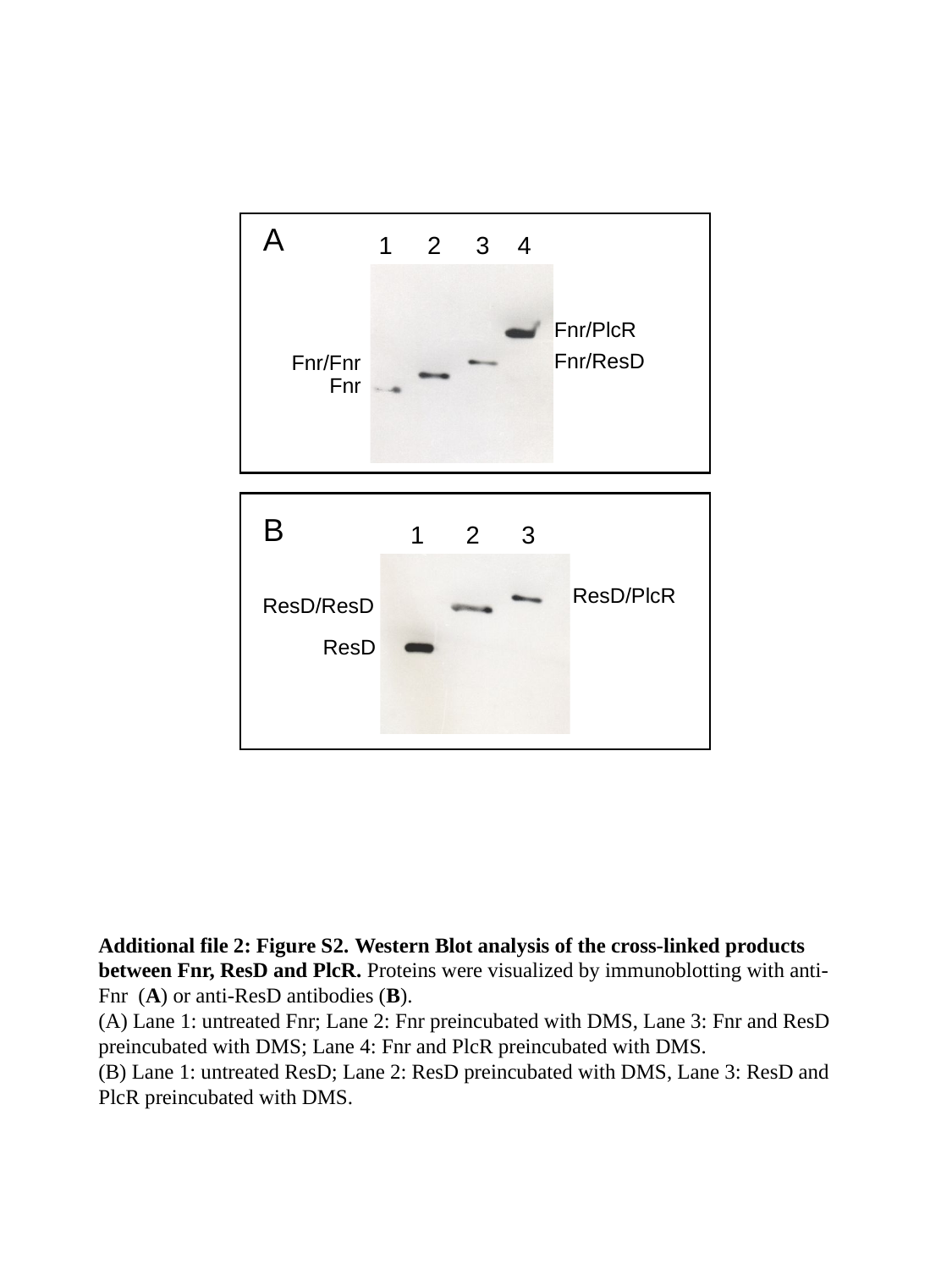

A
1 2 3 4
Fnr/PlcR
Fnr/ResD
Fnr/Fnr
Fnr
B
1 2 3
ResD/PlcR
ResD/ResD
ResD
Additional file 2: Figure S2. Western Blot analysis of the cross-linked products between Fnr, ResD and PlcR. Proteins were visualized by immunoblotting with anti-Fnr (A) or anti-ResD antibodies (B).
(A) Lane 1: untreated Fnr; Lane 2: Fnr preincubated with DMS, Lane 3: Fnr and ResD preincubated with DMS; Lane 4: Fnr and PlcR preincubated with DMS.
(B) Lane 1: untreated ResD; Lane 2: ResD preincubated with DMS, Lane 3: ResD and PlcR preincubated with DMS.
