# Supplementary material for: Bacillus cereus Fnr binds a [4Fe-4S] cluster and forms a ternary complex with ResD and PlcR
Source: BMC Microbiol. 2012 Jun 25;12:125. doi: 10.1186/1471-2180-12-125 (PMC3520743; doi:10.1186/1471-2180-12-125)
Supplement: Additional file 3 — Figure S3. Sequence analysis of B. cereus Fnr. Sequence alignment was performed using ClustalW software. Conserved residues are indicated by a star; conservatively substituted residues are indicated by a colon and semi-conservatively substituted residues are indicated by a point. The cysteine residues are indicated in bold. The cysteine residues 227, 230 and 235 that coordinate the [4Fe-4S]2+ cluster with aspartate residue 141 in B. subtilis are indicated in gray. [file 1471-2180-12-125-S3.pptx]

## Slide 1
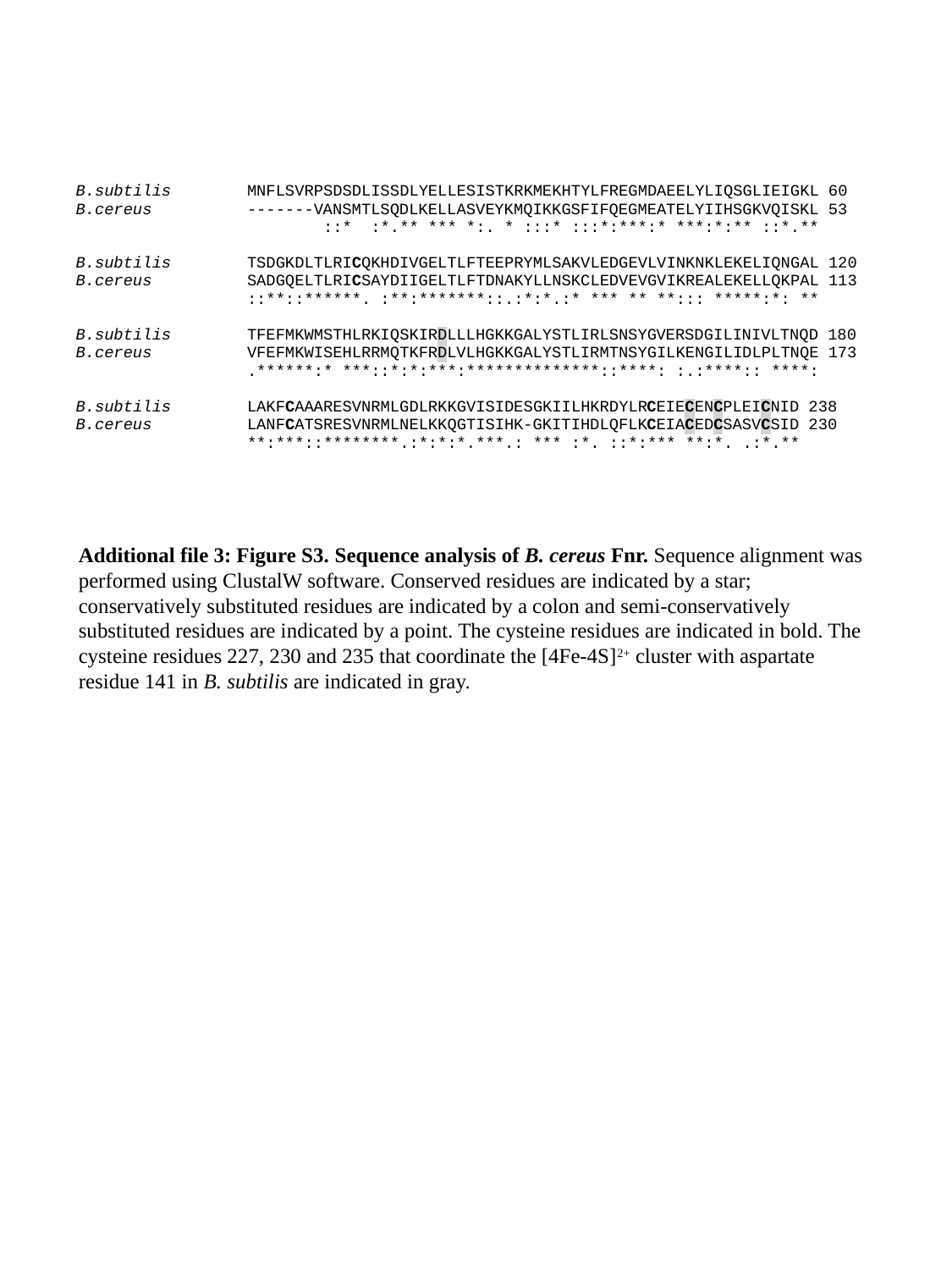

Additional file 3: Figure S3. Sequence analysis of B. cereus Fnr. Sequence alignment was performed using ClustalW software. Conserved residues are indicated by a star; conservatively substituted residues are indicated by a colon and semi-conservatively substituted residues are indicated by a point. The cysteine residues are indicated in bold. The cysteine residues 227, 230 and 235 that coordinate the [4Fe-4S]2+ cluster with aspartate residue 141 in B. subtilis are indicated in gray.
